# COVID-19 vaccines: anaphylaxis and anxiety: A case study from an allergy unit

**DOI:** 10.1007/s00508-024-02435-0

**Published:** 2024-09-11

**Authors:** Andrea R. Teufelberger, Andrada-Renata Dan, Linda Irmler, Peter Wolf, Birger Kränke

**Affiliations:** Department of Dermatology and Venereology, Medical University of Graz, Auenbruggerplatz 8, 8036 Graz, Austria

**Keywords:** Anaphylactoid, Polyethylene glycol, Questionnaire, Antihistamine, Intradermal test

## Abstract

**Background:**

Vaccination against severe acute respiratory syndrome coronavirus type 2 (SARS-CoV-2) was one crucial element to overcome the coronavirus disease 2019 (COVID-19) pandemic. Even though anaphylaxis to vaccines is rare, 47 patients came to the Allergy Unit at the University Hospital Graz, Austria, reporting immediate anaphylactoid symptoms after administration of COVID-19 vaccines. In addition, 29 patients with known drug-induced anaphylaxis wanted to be tested for a possible sensitization against COVID-19 vaccines or excipients, such as polyethylene glycol (PEG) or polysorbate 80 (PS80) before the first COVID-19 vaccination. Skin prick tests and intradermal tests were performed in all 76 patients, mostly using PEG 2000, and/or PS80. Skin prick tests with COVID-19 vaccines were performed depending on availability.

**Objective:**

Our aim was to characterize this patient cohort in terms of patients’ anaphylactoid responses, their willingness to future vaccinations against SARS-Cov‑2, and reasons for their decision.

**Methods:**

We developed a questionnaire and analyzed 34 completed copies.

**Results:**

Of the 47 patients with anaphylactoid reactions to COVID-19 vaccination, most were female (40 female/7 male). The skin tests, even when performed with the respective COVID-19 vaccine, were negative in all but one patient. Most patients who experienced anaphylactoid reactions after a COVID-19 vaccination, did not want another COVID-19 vaccination at the time of answering the questionnaire because of anxiety for another anaphylactoid response at the next shot. Premedication with antihistamines significantly lowered (*n* = 74 vaccinations) the severity of anaphylactoid responses after COVID-19 vaccinations.

**Conclusion:**

Anxiety about another anaphylactoid episode hinders patients to be vaccinated against SARS-CoV‑2 again. Premedication with antihistamines and collaboration of allergologists with psychologists might lower the risk of an anaphylactic/anaphylactoid response as well anxiety in drug-induced anaphylactic patients.

**Supplementary Information:**

The online version of this article (10.1007/s00508-024-02435-0) contains supplementary material, which is available to authorized users.

## Introduction

Shortly after the start of big scale coronavirus disease 2019 (COVID-19) vaccinations in late 2020, to overcome the corona pandemic, first cases of anaphylaxis have been reported. Overall, anaphylaxis to COVID-19 vaccines is rare and a strong sex bias towards women has been described by several studies [1, 2]. The agents triggering anaphylaxis in the COVID-19 vaccines have not been fully identified but based on previously described sensitizations to polyethylene glycol (PEG) or polysorbate 80 (PS80), these excipients have been the main suspects [3]. The Pfizer-BioNTech vaccine contains a novel pegylated lipid nanoparticle with a molecular weight of 2000 Dalton, abbreviated to PEG 2000, while the Moderna mRNA vaccine includes a different pegylated lipid (1,2-dimyristoyl-rac-glycero3-methoxypolyethyleneglycol-2000, PEG 2000-DMG), also a PEG 2000 [4]. As PEGs were previously considered to be biologically inert, this group of polyether compounds is widely used in medicinal, cosmetic and household products. Immediate type allergy to PEGs has a very low but increasing frequency, in the literature mostly reported due to high molecular weight PEGs [5, 6]. The mechanisms which underly PEG allergy are yet unclear, even though IgE antibodies to PEG have been detected in some patients with a history of PEG-induced anaphylaxis. In addition, PEGs have also been shown to induce complement activation in vitro and may therefore result in complement activation-related pseudo-allergy (CARPA) [4]. Polysorbates are derived from PEGs but tend to be of lower molecular weight: PS80 has a molecular weight of 1310 Dalton. Some of the current non-mRNA-based COVID-19 vaccines contain PS80, but in contrast to PEG 2000, PS80 is an excipient in many existing vaccines (e.g., Diphteria-Tetanus-Pertussis vaccine, Hepatitis B vaccine, Human Papilloma Virus vaccine, pneumococcal conjugate vaccine, influenza vaccines, zoster vaccine) [4]. Cross reactivity between PEGs and PS80 is still a matter of debate.

The skin prick test and intradermal test are the main skin tests used to determine sensitization for allergens; however, the reliability in the context of sensitization to COVID-19 vaccines has been questioned and seems controversial [7, 8]. The PEG 4000, also known as macrogol, is typically used in laxatives in the course of colonoscopy. A case report suggested a possible sensitization to PEG due to the COVID-19 vaccination in three cases, which led to an anaphylactic response to laxatives for the colonoscopy [9].

In this study, we characterized our cohort comprising drug-induced anaphylaxis patients and patients who reported anaphylactoid symptoms immediately after at least one of the COVID-19 vaccinations. In a questionnaire, we asked about their reactions immediately after vaccination, their possible pre-exposures to vaccine excipients, as well as their opinion and feelings concerning COVID-19 vaccinations.

## Patients, material and methods

### Baseline data

A total of 47 patients came to the Allergy Unit of the Department of Dermatology and Venereology at the Medical University Hospital Graz, Austria, reporting immediate anaphylactoid symptoms after administration of COVID-19 vaccines (Table 1). Of the patients 85.1% were female, which is in line with previous reports [2]. In addition, 29 patients with a history of drug-induced anaphylaxis wanted to be tested for sensitization against COVID-19 vaccines or vaccine excipients (PEG 2000, PS80) before their first vaccination.

**Table 1:** Reported symptoms at the consultation in our clinic by 47 patients, who received a coronavirus disease 2019 (COVID-19) vaccine and reported immediate anaphylactoid reactions

Symptoms	(*n*)
*Cardiovascular system*	
Vertigo	14
Dyspnea	11
Shivers	8
Tachycardia	7
Blood pressure rise	6
Blood pressure drop	6
Hot flush	6
Unconsciousness	3
Chest tightness	2
Difficulties to breathe	1
Circulatory problems	1
Almost blacking out	1
*Skin*	
Exanthem	11
Urticaria	2
Pruritus	10
Flush	5
*Paresthesia*	
Paresthesia (lips)	8
Paresthesia (mouth)	7
Paresthesia (palms and soles)	3
Paresthesia (feet)	2
Paresthesia (head and torso)	1
Paresthesia (vaccinated arm)	1
Paresthesia (whole body)	1
“Current pulses” (whole body)	1
*Gastrointestinal system*	
Nausea	8
Malaise	4
Vomiting	3
*Edema*	
Globus sensation	11
Swollen lips	7
Swollen tongue	3
Dysphagia	3
Swollen face and throat	1
Swollen hands	1
Sensation of pressure in mouth	1
*Other*	
Metallic taste in mouth	3
Eyes that glaze over	1
Myalgia	1
Cephalalgia	1
Burning sensation in hands and feet	1

*n* number of symptoms mentioned; several symptoms per patient are possible

Skin prick tests and intradermal tests were performed in all 76 patients using 10% PEG 2000 (ROTIPURAN Ph. Eur.; Carl Roth GmbH + Co. KG; Karlsruhe, Germany) (*n* = 63) and/or 0.5% PS80 (Polysorbitanum 80 Oleinatum; Gatt-Koller GmbH, Absam, Austria) (*n* = 27) sterile solutions, provided inhouse by the local hospital pharmacy. Furthermore, COVID-19 vaccines from BioNTech/Pfizer (*n* = 33; Mainz, Germany/New York, NY, USA), Astra Zeneca (*n* = 1; Cambridge, England), or Moderna (*n* = 10; Cambridge, MA, USA) were used for skin prick tests depending on availability and the vaccine brand, which was suspected to have triggered the anaphylactoid reaction. Skin prick tests with PEG 2000 solution, PS80 solution, and COVID-19 vaccines were performed undiluted. Intradermal tests with PEG and PS80 solutions were performed at 1:10 and 1:100 dilutions. In all but one patient the test results were negative, which was in line with previous studies [1, 10, 11]. The female individual with the positive intradermal test result was tested for PEG 2000 and PS80 (skin prick test and intradermal test). She responded with a positive local reaction at all four sites of the intradermal test. In addition, she was tested by skin prick test with the COVID-19 vaccine from BioNTech/Pfizer (skin prick test undiluted) and responded with a 1 mm wheal. In 2008, this patient was also tested with intradermal tests for the contrast agents Hexabrix, Ultravist, Iomeron, Xenetix and Visipaque and responded positively to Hexabrix and Ultravist.

Patients with a negative test result were given the recommendation for (repeated) COVID-19 vaccination and to take an antihistamine approximately 1h before the next COVID-19 vaccination.

### Study design

**Fig. 1:**
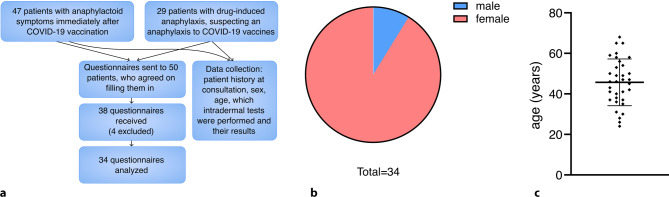
Overview of the study and general participant characteristics. **a** Study layout, **b** sex distribution and **c** age distribution of the 34 study participants. *COVID-19* coronavirus disease 2019

An overview of this study is depicted in Fig. 1a. We developed a questionnaire for this patient cohort (sent out in September 2022) to gather follow-up information. Inclusion criteria were ≥ 18 years of age and at least one anaphylactic or anaphylactoid reaction to medication and/or a COVID-19 vaccine (anamnestically diagnosed and graded according to Ring and Messmer grades 1–4) [12]. Exclusion criteria were inability to personally consent and a bad general state of health. The original German and translated questionnaire can be found in the supplement. In short, the questionnaire addressed reactions to COVID-19 vaccines, potential premedication, and sensitization, additional triggers of anaphylaxis, as well as the willingness and motives concerning future vaccinations. From the 76 patients, 52 (68%) were contacted and asked to fill in the questionnaire because they had a doctor’s rated anaphylaxis grade II or higher to a COVID-19 vaccine or to medication and 50 patients agreed to fill in the questionnaire. Of the patients 38 (74%) returned the filled-in questionnaires along with their written informed consent, 4 questionnaires were excluded from the analysis: 2 questionnaires contained conflicting statements, as the indicated answers did not align with the doctor’s notes. A third participant stated having actually experienced the symptoms 5 days after vaccination instead of an immediate response. The fourth participant was in the 13th week of gestation at the time of vaccination, and experienced nausea, headache, vertigo, paresthesia in lips and tongue, and tremor. Hence, we could not exclude that the symptoms might have been due to the pregnancy. This study was approved by the local ethical committee (ethical approval number: 34-467 ex 21/22) and all procedures were performed after written informed consent of the study participants in accordance with the ethical standards of the responsible committee on human experimentation and with the Helsinki Declaration of 1946, as revised in 2008 [13].

## Results

### Anaphylactoid responses after COVID-19 vaccines were less severe when antihistamines were taken as premedication

**Fig. 2:**
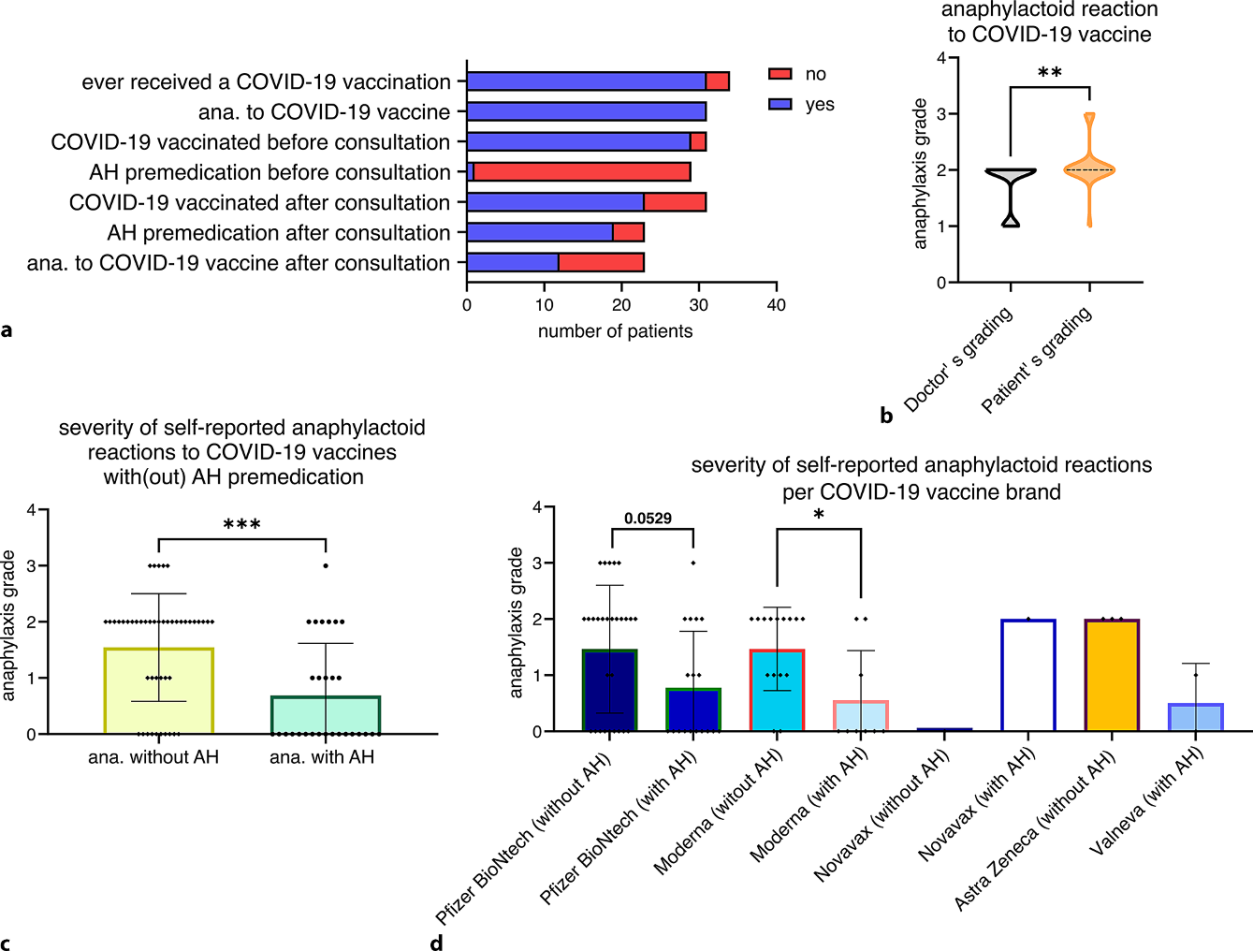
Anaphylactoid reactions after coronavirus disease 2019 (COVID-19) vaccinations. **a** Anaphylactoid reactions to COVID-19 vaccination and premedication before and after consultation, **b** comparison of doctor’s and patient’s anaphylaxis grading for COVID-19 vaccinations before consultation, **c** severity of self-reported anaphylactic reactions to COVID-19 vaccines of all vaccinations received by the study participants, without versus with AH premedication. Group comparison was performed using non-parametric Mann-Whitney test; the *P*-value is indicated, **d** data of **c** broken down per vaccine brand. *ana.* Anaphylaxis or anaphylactoid reaction, *AH* antihistamine

Out of 34 (91%) study participants 31 were female (Fig. 1b). The age ranged between 24 and 68 years (Fig. 1c). Of the 29 patients with drug-induced anaphylaxis who had come to the clinic to predict a sensitization to COVID-19 vaccine ingredients, 5 filled in the questionnaire and 3 did not receive a COVID-19 vaccination (Fig. 2a). One out of them was the patient, who had a positive intradermal test result. All study participants, who did receive at least one COVID-19 vaccination (31 patients), reported an anaphylactoid response shortly after at least one of their received COVID-19 vaccinations (Fig. 2a). In the questionnaire, we asked for the vaccine brand and at which vaccination time point antihistamines were taken as premedication and how strong the anaphylactic response was. A symptom table with grades according to Ring and Messmer was included in the questionnaire, to facilitate self-grading of the perceived anaphylactic reaction [12]. While only one patient already took antihistamines as premedication for their COVID-19 vaccination before the doctor’s appointment, most (68%) took antihistamines according to the doctor’s advice at their subsequent COVID-19 vaccination (Fig. 2a). Still, half of the patients who had at least one more shot of COVID-19 vaccine after the doctor’s consultation reported a (repeated) anaphylactoid response to it (Fig. 2a). To validate the patient’s grading, we compared the responses, which happened before the consultation with the doctor’s grading based on the anamnesis (Fig. 2b). The study participants tended to grade their anaphylactoid responses more severe than the medical doctor (Fig. 2b); however, when comparing their responses with or without antihistamine pretreatment, an overall significant reduction in severity was reported for COVID-19 vaccinations, when antihistamines were taken (Fig. 2c). This pattern recurred in different COVID-19 vaccine brands, where more than three vaccinations were reported (Fig. 2d). Of note, all but two vaccinations were carried out before September 2022. In September 2022, variant-adapted modifications of the vaccines became available in Austria. We can therefore exclude that the milder responses with antihistamine pretreatment are due to differences in the administered vaccines of the same brand.

### Most patients who reported an anaphylactoid reaction towards COVID-19 vaccines also reported anaphylactoid reactions to other triggers

**Fig. 3:**
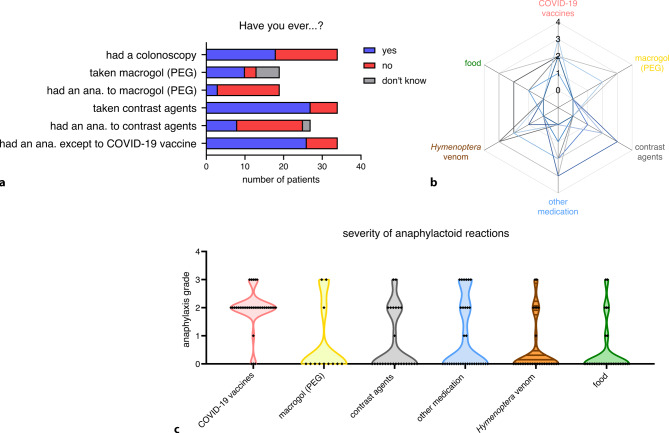
Potential sensitizations to polyethylene glycol (PEG), polysorbate 80 (PS80) or other triggers. **a** Evaluation of pre-exposures and additional anaphylactoid experience, **b** radar graph indicating study participants with possible polysensitization, **c** additional triggers of anaphylactoid reactions reported by the study participants. *ana.* anaphylaxis or anaphylactoid reaction, *COVID-19* coronavirus disease 2019

We wanted to know whether a possible PEG exposure from other medications, such as laxatives and ultrasound contrast agents, could have been associated with the reported anaphylactic response to COVID-19. Some but not all study participants likely had contact with ingredients such as high molecular weight PEG or PS80 prior to their COVID-19 vaccination (Fig. 3a). This might have led to a sensitization, although only 11 (32.3%) reported anaphylaxis to macrogol and/or contrast agents (Fig. 3a). Interestingly, 23 of the 31 (74%) COVID-19 vaccinated study participants reported to have experienced anaphylactoid reactions to triggers other than the COVID-19 vaccine (Fig. 3a), which has also previously been reported [14]. Of the patients 14 mentioned drug-induced anaphylaxis and some participants stated reactions to multiple triggers (Fig. 3b, c).

### Anaphylactoid reactions to COVID-19 vaccines caused anxiety to experience a similar response at the next COVID-19 vaccination, which correlated with refusal to vaccinate against COVID-19 in the future

**Fig. 4:**
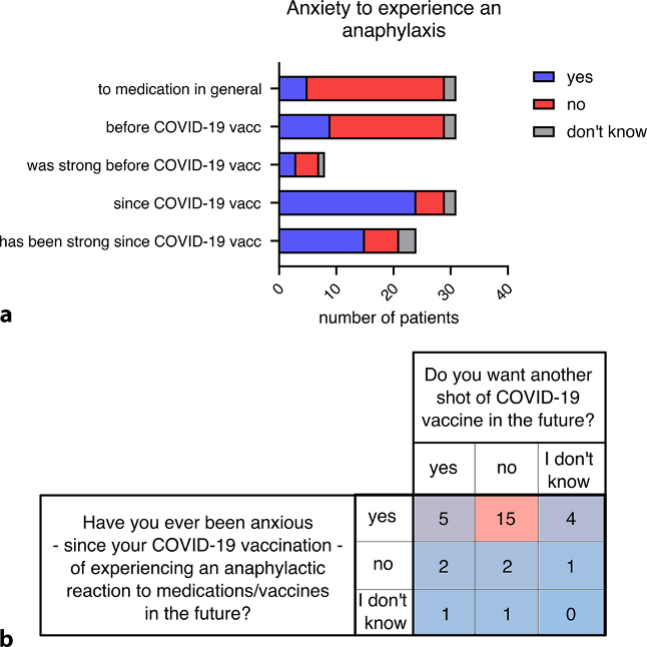
Anxiety and willingness concerning future coronavirus disease 2019 (COVID-19) vaccination (vacc). **a** Anxiety to experience an anaphylaxis in general, before they had their reaction to COVID-19 vaccination, since their reaction to COVID-19 vaccination and whether anxiety is strong, **b** correlation of anxiety to experience another anaphylactic or anaphylactoid episode and willingness to vaccinate against COVID-19 in the future

We wanted to know whether the experienced response to the COVID-19 vaccine triggered anxiety and whether this led to trigger avoidance. The study participants reported whether they had been anxious of an anaphylactic response to COVID-19 vaccines or other medication already before their reaction to the COVID-19 vaccine and/or since their reaction. We further asked if this fear was strong (Fig. 4a). As expected, more study participants have been anxious since their reaction to the COVID-19 vaccine (24 of 31; 77%), than before it (29%). Of those who were anxious since their reaction, 62.5% reported a strong feeling of anxiety for a future anaphylactic reaction upon COVID-19 vaccination (Fig. 4a). This seems mirrored in the high rate of 58% of refusal to be vaccinated against COVID-19 in the future. Indeed, when correlating these two parameters, most participants, who did not want another shot of COVID-19 vaccine in the future, reported anxiety of an anaphylactic reaction to it (Fig. 4b). The reasons given against another COVID-19 vaccination in the future partially indicated the same trend (Table 2): “to protect myself. The vaccine is not good for my body” and “due to my risk-benefit analysis” strongly indicate a comparable argumentation, as “I don’t want to experience an anaphylactic reaction again”. A total of 6 participants (19.3%) also reported to “still suffer from health problems due to that vaccine”; however, the eight participants, who were willing to have another COVID-19 vaccination in the future, had the completely opposite view as they saw the benefits of disease protection by the vaccination outweighing the risks of an anaphylactic reaction to the vaccine.

**Table 2:** Rationale given by the participants concerning their decision of future coronavirus disease 2019 (COVID-19) vaccination wish or refusal

Rationale: Do you want another COVID-19 vaccination in the future?	Not COVID-19 vaccinated (*n*)	COVID-19 vaccinated (*n*)
Yes—because the benefits outweigh the risks	–	6
Yes—because I can get immediate help in case of another anaphylactic reaction		2
No—to protect myself. This vaccine is not good for my body		3
No—due to my risk-benefit analysis	1	2
No—I don’t want to experience an anaphylactic reaction again	1	7
No—the initial immunization with three shots is enough	–	1
No—because I got COVID-19 despite my vaccination		1
No—because I am still suffering from health problems due to that vaccine		6
No (no reason given)		2
I don’t know—it depends on the availability of different COVID-19 vaccines		4
I don’t know—I want to wait for an adapted COVID-19 vaccine		1
I don’t know—maybe if it would be possible to be vaccinated with premedication and monitoring		1
I don’t know—test results at the hospital might influence my decision	1	–
I don’t know—because I got COVID-19 with a moderate course despite my vaccination	–	1

*n* number of reasons given; some patients gave several reasons

## Discussion

This is a case study from a small patient cohort in a single allergy center; however, despite the limited number of study participants and the intrinsically subjective answers of our questionnaire, we are confident to state that with this study, we gathered some interesting and important outcomes. First, we could confirm that a higher number of women were affected, and that intradermal tests with PEG 2000 and PS80 might not be suitable to predict an anaphylactic reaction to COVID-19 vaccines [1, 2, 7, 10, 11] as previously described in other studies. Negative skin test results do not rule out future anaphylaxis in a patient. By this, we are in accordance with previous reports that a pseudo-allergic response to COVID-19 vaccines or their excipients, as it can occur in drug-induced anaphylaxis, is conjectural [10, 15, 16].

Second, we could show that antihistamine premedication significantly decreased the severity of anaphylactoid reactions to COVID-19 vaccination in our study cohort. Only one study participant had a severe (grade 3) reaction despite premedication with antihistamines. In this case, it was indicated that the medication was taken 30 min before COVID-19 vaccination, which might have been too soon before the vaccination. All other anaphylactoid symptoms after COVID-19 vaccination with antihistamine premedication were either non-existing or at least not life-threatening. The reasoning of participants who wanted another COVID-19 vaccination in the future, was actually well in agreement with this observed ameliorating effect, as vaccinations with antihistamine premedication showed no or milder (grade ≤ 2) anaphylactic responses in our cohort. Antihistamine premedication should thus be recommended for patients with previous anaphylaxis. Another approach to avoid anaphylaxis or anaphylactoid reactions suggested by a medical center in Venice, was to divide vaccine injection into two parts with a waiting period of 20 min between them. This approach did not trigger anaphylactic reactions even in patients with diagnosed PEG sensitization [17].

Third, most patients who reported immediate anaphylactoid reactions after COVID-19 vaccinations also reported experienced anaphylactic or anaphylactoid reactions to other triggers, such as drugs, *Hymenoptera* venom and/or food. This indicates that patients reacting with anaphylactoid symptoms to COVID-19 vaccines might have a tendency to react more easily to external triggers in general; however, this does not mean that patients with anaphylaxis triggered by food or *Hymenoptera* venom will necessarily react to a COVID-19 vaccine, because in that case the reported frequency of anaphylactoid reactions to COVID-19 vaccines would be much higher.

Hesitancy to vaccinate against COVID-19 is quite common in the general population. According to a survey of the general population in the United Kingdom, 16.6% were unsure whether they wanted to be vaccinated against COVID-19 and 11.7% were strongly hesitant; however, the main motivation for vaccination refusal was mistrust and not fear of an anaphylactic event [18]. An anaphylactic reaction can trigger panic and fear for life. This can lead to a subsequent posttraumatic stress disorder [19]. It is thus not surprising that we found anxiety for anaphylaxis reoccurrence at the next COVID-19 vaccination and refusal for future COVID-19 vaccinations in most participants of our study. In general, it seems difficult to diagnose anaphylaxis to COVID-19 vaccines as not all mentioned symptoms (Table 1) are typical for anaphylactic or anaphylactoid reactions in our cohort. We also do not have information about serum tryptase levels during the anaphylactic episode of our patients, which could serve as a diagnostic marker. A differential diagnose of a panic reaction could thus be possible; however, we do not want to ascribe this to our patients, especially, as most participants reported no anxiety for anaphylaxis before they had their first anaphylactoid reaction to a COVID-19 vaccine.

Although skin testing to vaccine and vaccine excipients (in the case of COVID-vaccines) seems to lack precision, as the mechanism of adverse reactions in most cases is less likely to be IgE-mediated or the quantity of PEG/PS80 in the vaccines is very low and therefore subthreshold to provoke a reaction, the other side of the coin is a reported sensitivity of 95.8% regarding skin tests in patients with suspected PEG allergy in a large review [6, 20, 21]. Therefore, testing vaccine excipients may be useful for detecting those who, with a personal history highly compatible with PEG/PS allergy, have developed a specific IgE reactivity, to address the consequent decision-making and estimate the risks of other possible PEG/PS exposures [20].

In conclusion, our results demonstrate the urgent need for studies focusing on the underlying immunological mechanisms of these reactions to learn how they can be diagnosed, predicted, and avoided. A clear diagnosis and appropriate measures of avoidance could reduce anxiety in those affected and might increase their willingness to be vaccinated (with antihistamine premedication) in the future. In addition, it might be useful to establish an interdisciplinary collaboration with psychologists to assist drug-induced anaphylaxis patients dealing with their anxiety.

## Supplementary Information

Supplement: Questionnaires in original language (German) and English
