# Supplementary material for: COVID-19 vaccines: anaphylaxis and anxiety: A case study from an allergy unit
Source: Wien Klin Wochenschr. 2024 Sep 11;136(21-22):590–7. doi: 10.1007/s00508-024-02435-0 (PMC11534977; doi:10.1007/s00508-024-02435-0)
Supplement: Supplementary file 1 — Supplement: Questionnaires in original language (German) and English [file 508_2024_2435_MOESM1_ESM.docx]

Title: **COVID-19 vaccines: anaphylaxis and anxiety - a case study from an allergy unit**

Titel: **COVID-19 Impfungen: Anaphylaxie und Angst – ein Erfahrungsbericht einer Allergieambulanz**

Supplement: Questionnaires in original language (German) and English.

**Fragebogen**

Fragebogen zur Studie „**Diplomarbeit zum Thema: Anaphylaktische Reaktion vom Soforttyp auf die COVID-19 Impfungen**“.

Sehr geehrte/r Studienteilnehmer/in!

Im Rahmen meiner Diplomarbeit an der Medizinischen Universität Graz forsche ich an anaphylaktischen Reaktionen auf COVID-19 Impfungen am LKH Graz, an der Universitätsklinik für Dermatologie und Venerologie. Es handelt sich eine Studie, welche Daten erforscht, um etwaige Zusammenhänge der anaphylaktischen Reaktionen zu charakterisieren welche sofort nach der COVID-19 Impfung aufgetreten sind. „Anaphylaxie“ ist das plötzliche Auftreten von schweren, körperlichen (=anaphylaktischen) Reaktionen, in diesem Fall auf die COVID-19 Impfung, die potenziell lebensbedrohlich sein können. Diese werden in unterschiedliche Schweregrade unterteilt (siehe unten).

Ich danke Ihnen sehr herzlich, dass Sie sich bereiterklärt haben, an dieser Studie teilzunehmen. Nun benötigen wir noch einige Informationen von Ihnen, welche im darauffolgenden Fragebogen abgefragt werden. Deshalb bitte ich Sie diese gut durchzulesen, auszufüllen und mit dem beigelegten bereits frankierten Kuvert zurückzuschicken. Die Datenauswertung erfolgt, wie bereits im Einverständnisbogen erklärt, unter Pseudonymisierung und strenger Geheimhaltung Ihrer Daten.

Daten zur Person:

**Nachname:**

**Vorname:**

**Geburtsdatum:**

**Geschlecht:**

Frage 1.)

Haben Sie schon mindestens einmal eine Darmspiegelung gehabt?

🞏 Ja 🞏 Nein 🞏 weiß nicht

1a.) Wenn ja: haben Sie dafür ein Mittel zur Darmentleerung, das „Macrogol“ enthält (wie Endofalk®, Klean-Prep®, Laxogol®, Molaxole®, Movicol®, Moviprep®, Olopeg®, Pleinvue®) eingenommen?

🞏 Ja 🞏 Nein 🞏 weiß nicht

1b.) Wenn ja, haben Sie darauf allergisch/anaphylaktisch reagiert?

🞏 Ja 🞏 Nein 🞏 weiß nicht

1c.) Wenn ja: wie stark?

Kreuzen Sie bitte mit Hilfe dieser Tabelle den Grad Ihrer erlittenen Reaktion bei dieser Frage und allen folgenden Fragen, bei denen die Gradeinteilung abgefragt wird, an. Kreuzen Sie bitte den höchsten Grad an, in dem Sie mindestens ein zutreffendes Symptom hatten. (z.B.: Sie hatten Juckreiz (Grad I), einen Hautausschlag (Grad I), Schwindelgefühl (Grad II) und mussten erbrechen (Grad III) -> Kreuzen Sie Grad III an, weil Sie erbrechen mussten. Oder Sie hatten eine Hautrötung (Grad I), Hautschwellung (Grad I), Herzrasen (Grad II) und beginnende Atemnot (Grad II) -> Kreuzen Sie Grad II, aufgrund des Herzrasens und der beginnenden Atemnot, an.)

| **Grad I** | **Grad II** | **Grad III** | **Grad IV** |
| --- | --- | --- | --- |
| Veränderungen an der Haut wie:  -Juckreiz  -Rötung  -Schwellung  -Hautausschlag | messbare, aber nicht lebensbedrohliche Reaktionen des Herz-Kreislaufsystems wie:  -Herzklopfen/rasen  -Blutdruckabfall, Schwindel  -Bauchschmerzen  -Übelkeit  -beginnende Atemnot  -Druckgefühl auf der Brust | lebensbedrohliche Zustände wie:  -Bewusstlosigkeit  -Atemnot  -Bauchkrämpfe  -Erbrechen  -blaue Verfärbung der Haut  -Inkontinenz | Atem- und/oder Kreislaufstillstand:  -vollkommener Atemstillstand  -hier ist eine Wiederbelebung (Reanimation) notwendig |

🞏 Grad I 🞏 Grad II 🞏 Grad III 🞏 Grad IV 🞏 weiß nicht

Frage 2.) Haben Sie schon einmal eine Untersuchung mit einem Kontrastmittel (CT, MRT) gehabt?

🞏 Ja 🞏 Nein 🞏 weiß nicht

2a.) Wenn ja: haben Sie darauf allergisch/anaphylaktisch reagiert?

🞏 Ja 🞏 Nein 🞏 weiß nicht

2b.) Wenn ja: wie stark (Gradeinteilung wie Tabelle oben)?

🞏 Grad I 🞏 Grad II 🞏 Grad III 🞏 Grad IV 🞏 weiß nicht

Frage 3.) Haben Sie generell, unabhängig von der COVID-19 Impfung schon einmal anaphylaktisch reagiert?

🞏 Ja 🞏 Nein 🞏 weiß nicht

3a.) Wenn ja: wie stark?

🞏 Grad I 🞏 Grad II 🞏 Grad III 🞏 Grad IV 🞏 weiß nicht

3b.) Wenn ja, was war(en) der/die Auslöser? (z.B. Bienen/Wespenstiche, Medikamente, Lebensmittel)

__________________________________________________________________________________

Frage 4.)

Haben Sie sich gegen COVID-19 impfen lassen?

🞏 Ja 🞏 Nein (bei Nein, bitte direkt mit Frage 7 weitermachen)

Frage 4a.) Wenn ja: mit welchen Impfstoffen (Pfizer/Moderna/AstraZeneca, etc.) wurden Sie wann bei welcher Teilimpfung geimpft? (nicht absolvierte Teilimpfung(en) bitte durchstreichen)

Impfstoff Datum

1.Teilimpfung______________________________________ _________________________________

2.Teilimpfung______________________________________ _________________________________

3.Teilimpfung______________________________________ _________________________________

4.Teilimpfung______________________________________ _________________________________

Frage 5.) Haben Sie auf mindestens eine Teilimpfung der COVID-19 Impfungen allergisch/anaphylaktisch reagiert?

🞏 Ja 🞏 Nein 🞏 weiß nicht

Frage 5a.) Wenn ja: auf die wievielte(n) Teilimpfung(en) haben Sie reagiert?

__________________________________________________________________________________

Frage 5b.) Wenn ja, wie stark (Gradeinteilung siehe Tabelle oben) haben Sie reagiert?

1. Teilimpfung: 🞏Grad I 🞏 Grad II 🞏 Grad III 🞏 Grad IV 🞏 weiß nicht 🞏 nicht reagiert

2. Teilimpfung: 🞏Grad I 🞏 Grad II 🞏 Grad III 🞏 Grad IV 🞏 weiß nicht 🞏 nicht reagiert

3. Teilimpfung: 🞏Grad I 🞏 Grad II 🞏 Grad III 🞏 Grad IV 🞏 weiß nicht 🞏 nicht reagiert

4. Teilimpfung: 🞏Grad I 🞏 Grad II 🞏 Grad III 🞏 Grad IV 🞏 weiß nicht 🞏 nicht reagiert

Frage 6.) Haben Sie in den Stunden **vor** (mindestens einer) Ihrer COVID-19 Teilimpfung(en) ein Antihistaminikum oder ein anderes Medikament eingenommen?

🞏 Ja 🞏 Nein 🞏 weiß nicht

Frage 6a.) Wenn ja: welches, wie lange vor welcher Ihrer Teilimpfungen und wie viel (Dosierung)?

1.Teilimpfung_______________________________________________________________________

2.Teilimpfung_______________________________________________________________________

3.Teilimpfung_______________________________________________________________________

4. Teilimpfung_______________________________________________________________________

Frage 7.) Hatten/Haben Sie vor Ihrer ersten COVID-19 Impfung Sorgen/Angst davor, auf diese allergisch/anaphylaktisch zu reagieren?

🞏 Ja 🞏 Nein 🞏 weiß nicht

Frage 8.) Haben und/oder hatten Sie generell – auch schon vor Ihrer Reaktion auf die COVID-19 Impfung(en) – Sorgen/Angst, dass Sie durch die Einnahme von Medikamenten bzw. anderen Impfungen (NICHT COVID-19) eine anaphylaktische Reaktion erleiden?

🞏 Ja 🞏 Nein 🞏 weiß nicht 🞏 habe nicht anaphylaktisch auf die Impfung reagiert

🞏 habe zwar nicht anaphylaktisch auf die Impfung reagiert, aber habe Angst vor anaphylaktischen Reaktionen auf andere Medikamente oder Impfungen

Frage 8a.) Wenn ja oder der letzte Punkt zutreffen: war Ihre Sorge/Angst groß?

🞏 Ja 🞏 Nein 🞏 weiß nicht

Frage 9.) Haben Sie **seit** der COVID-19 Impfung Sorgen/Angst, dass sie erneut allergisch/anaphylaktisch auf andere Medikamente und Impfungen reagieren könnten?

🞏 Ja 🞏 Nein 🞏 weiß nicht 🞏 nicht zutreffend, da nicht geimpft

Frage 9a.) Wenn ja oder nicht geimpft: ist Ihre Sorge oder Angst groß?

🞏 Ja 🞏 Nein 🞏 weiß nicht

Frage 10.) Möchten Sie sich in Zukunft mit einer weiteren COVID-19 Teilimpfung impfen lassen?

🞏 Ja 🞏 Nein 🞏 weiß nicht

Frage 10a.) Welche Überlegungen spielen bei dieser Entscheidung mit?

__________________________________________________________________________________

__________________________________________________________________________________

__________________________________________________________________________________

Frage 11.) Möchten Sie uns noch etwas zum Thema COVID-19 Impfungen, Arzneimittelreaktionen und Anaphylaxie mitteilen?

__________________________________________________________________________________

**Questionnaire**

Questionnaire for the study **"Diploma thesis on the topic: Immediate-type anaphylactic reaction to COVID-19 vaccinations".**

Dear study participant!

As part of my diploma thesis at the Medical University of Graz, I am researching anaphylactic reactions to COVID-19 vaccinations at the University Clinic for Dermatology and Venereology, University Hospital Graz. It is a study exploring data to characterize any associations of anaphylactic reactions that occurred immediately following COVID-19 vaccination. "Anaphylaxis" is the sudden onset of severe, physical (=anaphylactic) reactions, in this case to COVID-19 vaccination, that can be potentially life-threatening. These are divided into different grades of severity (see below).

Thank you very much for agreeing to participate in this study. Now we need some more information from you, which will be asked in the following questionnaire. Therefore, I would like to ask you to read it carefully, fill it in and send it back to us in the enclosed pre-stamped envelope. The data evaluation is carried out under pseudonymization and strict secrecy of your data, as already explained in the informed consent form.

personal data:

**surname:**

**first name:**

**date of birth:**

**sex:**

Question 1.)

Have you ever had at least one colonoscopy?

🞏 Yes 🞏 No 🞏 I don’t know

1a.) If yes: Have you taken a bowel evacuation remedy containing „Macrogol“ (like Endofalk®, Klean-Prep®, Laxogol®, Molaxole®, Movicol®, Moviprep®, Olopeg®, Pleinvue®)?

🞏 Yes 🞏 No 🞏 I don’t know

1b.) If yes, did you experience an allergic/anaphylactic reaction to it?

🞏 Yes 🞏 No 🞏 I don’t know

1c.) If yes: how strong?

Please tick the degree of reaction you suffered for this question and all subsequent questions that ask for the degree classification, with the help of this table. Please tick the highest degree in which you had at least one applicable symptom. (For example: You had itching (grade I), a skin rash (grade I), dizziness (grade II), and had to vomit (grade III) -> Tick grade III because you had to vomit. Or you had a skin redness (grade I), skin swelling (grade I), heart palpitations (grade II), and a beginning shortness of breath (grade II) -> Tick grade II, because of the heart palpitations and beginning shortness of breath).

| **Grade I** | **Grade II** | **Grade III** | **Grade IV** |
| --- | --- | --- | --- |
| Changes in the skin such as:  -itching  -redness  -swelling  -rash | Measurable but not life-threatening cardiovascular reactions such as:  -heart palpitations/tachycardia  -blood pressure drop, dizziness  -abdominal pain  -nausea  -beginning of shortness of breath  -feeling of pressure on the chest | Life-threatening conditions such as:  -unconsciousness  -breathlessness  -abdominal cramps  -vomiting  -blue discoloration of the skin  -incontinence | Respiratory and/or circulatory arrest:  -complete respiratory arrest  -here reanimation is necessary |

🞏 Grade I 🞏 Grade II 🞏 Grade III 🞏 Grade IV 🞏 I don’t know

Question 2.) Have you ever had a medical examination with contrast agents (CT, MRI)?

🞏 Yes 🞏 No 🞏 I don’t know

2a.) If yes, did you experience an allergic/anaphylactic reaction to it?

🞏 Yes 🞏 No 🞏 I don’t know

2b.) If yes: how strong (grading according to the table above)?

🞏 Grade I 🞏 Grade II 🞏 Grade III 🞏 Grade IV 🞏 I don’t know

Question 3.) Have you ever, in general, experienced an anaphylaxis, independent of the COVID-19 vaccination?

🞏 Yes 🞏 No 🞏 I don’t know

3a.) If yes: how strong?

🞏 Grade I 🞏 Grade II 🞏 Grade III 🞏 Grade IV 🞏 I don’t know

3b.) If yes, what were/was the trigger(s)? (e.g. bee/wasp stings, medication, food)

__________________________________________________________________________________

Question 4.)

Have you had a COVID-19 vaccination?

🞏 Yes 🞏 No (if no, please directly go on with question 7)

Question 4a.) If yes: with which vaccine brand (Pfizer/Moderna/AstraZeneca, etc.) have you gotten which COVID-19 vaccination and when? (please scratch through vaccination you did not have)

Vaccine brand Date of vaccination

1^st^ vaccination_____________________________________ _________________________________

2^nd^ vaccination_____________________________________ _________________________________

3^rd^ vaccination_____________________________________ _________________________________

4^th^ vaccination_____________________________________ _________________________________

Question 5.) Have you reacted with an allergic/anaphylactic response to at least one COVID-19 vaccination?

🞏 Yes 🞏 No 🞏 I don’t know

Question 5a.) If yes: to which of your vaccinations did you react?

__________________________________________________________________________________

Question 5b.) If yes, how strong did you react (grading according to the aforementioned table)?

1^st^ vaccination: 🞏 Grade I 🞏 Grade II 🞏 Grade III 🞏 Grade IV 🞏 I don’t know 🞏 I had no reaction

2^nd^ vaccination: 🞏 Grade I 🞏 Grade II 🞏 Grade III 🞏 Grade IV 🞏 I don’t know 🞏 I had no reaction

3^rd^ vaccination: 🞏 Grade I 🞏 Grade II 🞏 Grade III 🞏 Grade IV 🞏 I don’t know 🞏 I had no reaction

4^th^ vaccination: 🞏 Grade I 🞏 Grade II 🞏 Grade III 🞏 Grade IV 🞏 I don’t know 🞏 I had no reaction

Question 6.) Have you taken an antihistamine or any other medication within hours **before** (at least one hour upfront) at least one of your COVID-19 vaccinations?

🞏 Yes 🞏 No 🞏 I don’t know

Question 6a.) If yes: which medication, how long prior to which of your vaccinations, and how much (dosage)?

1^st^ vaccination______________________________________________________________________

2^nd^ vaccination______________________________________________________________________

3^rd^ vaccination______________________________________________________________________

4^th^ vaccination______________________________________________________________________

Question 7.) Are you/were you anxious before your first COVID-19 vaccination to react to it with allergic/anaphylactic symptoms?

🞏 Yes 🞏 No 🞏 I don’t know

Question 8.) Are you/were you anxious in general – also already before your reaction to COVID-19 vaccination(s) – to experience an anaphylactic reaction by taking medication or other vaccinations (NOT COVID-19 vaccination)?

🞏 Yes 🞏 No 🞏 I don’t know 🞏 I did not react with anaphylaxis to the vaccination

🞏 I did not react with anaphylaxis to the vaccination, but I am afraid of anaphylactic reactions to other medications and vaccinations

Question 8a.) If yes, or the last option apply: Is your anxiety strong?

🞏 Yes 🞏 No 🞏 I don’t know

Question 9.) Have you been anxious since your reaction(s) to the COVID-19 vaccination(s) to react to it and other medication or vaccinations with allergic/anaphylactic symptoms in the future?

🞏 Yes 🞏 No 🞏 I don’t know 🞏 not applicable, because not vaccinated

Question 9a.) If yes or not vaccinated: Is your anxiety strong?

🞏 Yes 🞏 No 🞏 I don’t know

Question 10.) Do you want another shot of COVID-19 vaccination in the future?

🞏 Yes 🞏 No 🞏 I don’t know

Question 10a.) Which considerations influence your decision?

__________________________________________________________________________________

__________________________________________________________________________________

__________________________________________________________________________________

Question 11.) Do you want to tell us something else concerning the topics COVID-19 vaccinations, reaction to drugs, and anaphylaxis?

__________________________________________________________________________________
